# Health Outcomes and Cost Impact of the New WHO 2013 Guidelines on Prevention of Mother-to-Child Transmission of HIV in Zambia

**DOI:** 10.1371/journal.pone.0090991

**Published:** 2014-03-06

**Authors:** Naoko Ishikawa, Takuro Shimbo, Shinsuke Miyano, Izukanji Sikazwe, Albert Mwango, Massimo N. Ghidinelli, Gardner Syakantu

**Affiliations:** 1 National Center for Global Health and Medicine, Tokyo, Japan; 2 Centre for Infectious Disease Research in Zambia, Lusaka, Zambia; 3 Ministry of Health, Lusaka, Zambia; 4 Pan American Health Organization, Washington, D.C., United States of America; 5 Ministry of Health, Lusaka, Zambia; UCL Institute of Child Health, University College London, United Kingdom

## Abstract

**Background:**

Countries are currently progressing towards the elimination of new paediatric HIV infections by 2015. WHO published new consolidated guidelines in June 2013, which now recommend either ‘Antiretroviral drugs (ARVs) for women living with HIV during pregnancy and breastfeeding (Option B)’ or ‘Lifelong antiretroviral therapy (ART) for all pregnant and breastfeeding women living with HIV (Option B+)’, while de facto phasing out Option A. This study examined health outcomes and cost impact of the shift to WHO 2013 recommendations in Zambia.

**Methods:**

A decision analytic model was developed based on the national health system perspective. Estimated risk and number of cases of HIV transmission to infants and to serodiscordant partners, and proportions of HIV-infected pregnant women with CD4 count of ≤350 cells/mm^3^ to initiate ART were compared between 2010 Option A and the 2013 recommendations. Total costs of prevention of mother-to-child transmission of HIV (PMTCT) services per annual cohort of pregnant women, incremental cost-effectiveness ratio (ICER) per infection averted and quality-adjusted life-year (QALY) gained were examined.

**Results:**

Our analysis suggested that the shift from 2010 Option A to the 2013 guidelines would result in a 33% reduction of the risk of HIV transmission among exposed infants. The risk of transmission to serodiscordant partners for a period of 24 months would be reduced by 72% with ‘ARVs during pregnancy and breastfeeding’ and further reduced by 15% with ‘Lifelong ART’. The probability of HIV-infected pregnant women to initiate ART would increase by 80%. It was also suggested that while the shift would generate higher PMTCT costs, it would be cost-saving in the long term as it spares future treatment costs by preventing infections in infants and partners.

**Conclusion:**

The shift to the WHO 2013 guidelines in Zambia would positively impact health of family and save future costs related to care and treatment.

## Introduction

Human Immunodeficiency Virus (HIV) can be transmitted from infected mothers to their infants during pregnancy, labour, delivery, and breastfeeding period. The risk of transmission is 15–30% in non-breastfed infants and 20–45% in breastfed infants [1]. Antiretroviral (ARV) prophylaxis could effectively reduce the transmission risk to less than 5% in breastfed infants and to less than 2% in non-breastfed infants [2]. Currently countries are moving towards the elimination of new paediatric HIV infections by 2015 [3]. It is estimated that a total of 260,000 children were infected with HIV in 2012, whereas 670,000 perinatal infections were prevented in low- and middle-income countries between 2009 and 2012 [4].

The 2010 guidelines of the World Health Organization (WHO) for the prevention of mother-to-child transmission of HIV (PMTCT) recommended two options: Option A and Option B [2]. In Option A, zidovudine (ZDV) is provided to HIV-infected pregnant women during antepartum period followed by nevirapine (NVP) prophylaxis for their infants during breastfeeding period. In Option B, maternal triple ARV prophylaxis is initiated during pregnancy and continued throughout breastfeeding period. In both options, above mentioned prophylaxis is provided to HIV-infected pregnant women with CD4 cell count of above 350 cells/mm^3^, whereas antiretroviral therapy (ART) is provided for those with CD4 cell count of ≤350 cells/mm^3^. In April 2012, WHO issued a programmatic update which proposed a third option: Option B+, in which maternal triple ARV drugs are continued throughout life regardless of CD4 count based on suggested clinical and programmatic advantages of adopting single regimen for all women [5]. Some countries have already begun the process of shifting to Option B or B+. In June 2013, WHO published new consolidated guidelines on the use of antiretroviral drugs for treating and preventing HIV infection, which now recommend either ‘ARV drugs for women living with HIV during pregnancy and breastfeeding (2010 guidelines Option B)’ or ‘Lifelong ART for all pregnant and breastfeeding women living with HIV (2010 guidelines Option B+)’, while de facto phasing out Option A [6].

Provision of triple ARV drugs to HIV-infected pregnant women is important not only to prevent HIV transmission to their infants but also to extend prevention benefits to their HIV negative partners as well as to improve their own health. Possibility of increased risk of female-to-male HIV transmission during pregnancy in the absence of treatment [7] and reduced risk of transmission among serodiscordant couples through the early initiation of ART [8] strongly support the need for ARVs for HIV-infected pregnant women. Reduced adverse pregnancy outcomes by extended antenatal use of triple ARV [9] further underpins the benefits of provision of ARV drugs.

With an HIV prevalence among women aged 15 to 49 years of 16.1%, Zambia is one of the 22 priority countries of the Global Plan to eliminate new HIV infection among children by 2015 [3], [10]. Zambia adopted Option A in 2010 and has been making effort to further expand PMTCT services. In 2011, 96.7% of pregnant women who attended antenatal care were tested for HIV, 74.9% of HIV-infected pregnant women received ARV prophylaxis or ART based on Option A, and 35.8% of HIV-exposed infants received NVP prophylaxis [10]. Zambia has decided to shift from Option A to Option B+ in January 2013 and started the development of an implementation plan. This policy shift was underpinned by the operational complexity of Option A, such as different regimens during pregnancy, labour, and postpartum period as well as dosage adjustments for HIV-exposed infants according to their body weights, and also by the expected added-benefit of Option B/B+ including the initiation of ART for HIV positive women to improve their own health and the prevention of HIV transmission to potential HIV negative partners [11]. As of January 2014, an official announcement of the transition to Option B+ has been made and its preparation is ongoing including the site assessment and update of the national PMTCT guidelines.

With the aim of estimating the expected impact of this shift, this study compared 2010 guidelines Option A and the 2013 guidelines (Option B and B+) with regards to the effectiveness in preventing transmission to infants as well as to serodiscordant partners, and initiating ART for HIV-infected women. It also examined the cost implications, especially with regards to cost-effectiveness of the 2013 guidelines.

## Methods

### Model Overview

**Figure 1:**
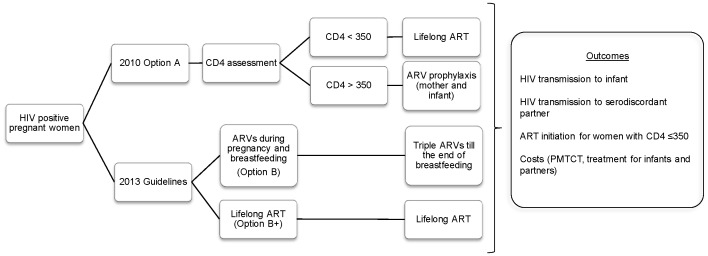
Model overview.

We developed a decision analytic model based on the national health system perspective and compared expected health outcomes and costs of 2010 Option A and 2013 guidelines: ARVs during pregnancy and breastfeeding (Option B) and Lifelong ART (Option B+) (Figure 1). The model started with an annual cohort of HIV-infected pregnant women in Zambia. In the model, it was assumed that all HIV-infected pregnant women were diagnosed HIV positive for the first time during the current pregnancy. Then as per 2010 Option A, CD4 assessment was provided and its results guided initiation of ART or ARV (ZDV) prophylaxis for women. In this analysis, CD4 cell count of ≤350 cells/mm^3^ represented the priority eligibility criterion for initiating ART. It was assumed that for those who could not access CD4 test and/or did not receive the results of the tests, ARV prophylaxis was provided. ARV prophylaxis for exposed infants was considered independently regardless of their mothers’ access to prophylaxis, since there had been cases of HIV-exposed infants identified for the first time after the delivery and started on prophylaxis. For ARVs during pregnancy and breastfeeding (Option B) and Lifelong ART (Option B+), triple ARV drugs were provided without CD4 assessment. We then estimated the probability of HIV transmission to exposed infants and to serodiscordant partners, and the probability of ART initiation for HIV-infected pregnant women with CD4 cell count ≤350 cells/mm^3^ for each option. TreeAge Pro 2012 (TreeAge Software, Inc.) was used for the development of the decision tree and the analysis.

### Model Inputs

Model inputs used in this study are shown in Table 1. Probabilities of perinatal HIV transmission to infants were based on the estimates by UNAIDS reference group on estimates, modelling and projections [12]. Probabilities of transmission to serodiscordant partners were estimated based on available evidence including HPTN 052 study [8], [13], [14], [15], [16]. We assumed that maternal ARV prophylaxis for 2010 Option A did not reduce the risk of HIV transmission to serodiscordant partners. CD4 cell count distribution of HIV-infected pregnant women was based on the study by Carter and others [17]. Discordance rate (i.e. HIV positive female aged 15–49 with HIV negative partner) was estimated at 36.8% based on the national data [18]. It was assumed that HIV-infected pregnant women initiated ARV prophylaxis or ART from the 14th week of pregnancy and HIV-exposed infants were breastfed until 12 months of age. The time horizon for the analysis was from the first antenatal care to 18 months after delivery. In addition, a time horizon of 10 years was also applied when the long-term cost implication of treatment for infected children and serodiscordant partners was examined. Demographic data and programme inputs for PMTCT were based on the UN data, Zambia national report 2012, and field level data collected by the Zambia Ministry of Health – Japan International Cooperation Agency scaling up of quality HIV/AIDS care service management project (SHIMA) in collaboration with National Center for Global Health and Medicine (NCGM) [10], [19].

**Table 1:** Model inputs.

Table 1a. Model inputs (health)		
Input	Value	Source (reference)
**Epidemiological parameters**		
Annual number of births	600,000	19
HIV prevalence (women aged 15–49)	16.1%	10
Discordance rate (HIV+ women aged 15–49 with HIV− partners)	36.8%	18
CD4 cell count distribution		
<350	45.4%	17
>350	54.6%	
**Perinatal HIV transmission**		
Peripartum period		
No prophylaxis CD4<350	27–37%	12
No prophylaxis CD4>350	15%	
Option A/B	2%	
ART	2%	
Postnatal period	(per month of breastfeeding)	
No prophylaxis CD4<350	1.57%	
No prophylaxis CD4>350	0.51%	
Option A/B	0.2%	
**HIV transmission to serodiscordant partner** (per 100 person-year)		
No ART CD4<350	9.2	8, 13–16
No ART CD4>350	1.7	
On ART CD4<350	0.7	
On ART CD4>350	0.1	
**Health services**		
Antenatal care attendance (at least once)	91.6%	10 & field data
HIV test among ANC attendees	96.7%	
Access to CD4 test	60.0%	
Received CD4 result	61.0%	
Initiation of maternal ARV prophylaxis	74.9%	
Initiation of maternal ART	47.0%	
Initiation of infant NVP prophylaxis	35.8%	
**Quality-adjusted life years (QALYs)**		
QALYs gained per infant infection averted	16.88	25
QALYs gained per partner infection averted	5.83	26
**Table 1b** **. Model inputs (cost)**		
**Input**	**Value**	**Source (reference)**
ARV prophylaxis	**USD**	
2010 Option A		estimated based on
Maternal ARV per course	47.6	20–23
Infant ARV for 12months	9.5	
ARVs during pregnancy and breastfeeding		
Maternal 3ARVs per course	260	
Infant ARV for 6weeks	1.9	
ART		
continue 12months postnatal	260	
continue 18months postnatal	345	
Laboratory test		
HIV rapid test	1	
CD4	5	
DNA PCR	10	
Viral load	28	
Health services (clinic visit)		
Health centre (per visit)	3.05	24
District hospital (per visit)	3.48	
Treatment for 10 years (drugs+laboratory monitoring+clinic visit)	20–24	
Child	1864	
Adult (TDF/FTC/EFV)	2370	

### Cost Inputs

Costs of PMTCT services and HIV treatment were estimated using the Costing Tool for Elimination Initiatives (CTEI) developed by NCGM in collaboration with Asia-Pacific United Nations Task Force for the Prevention of Parents-to-Child Transmission of HIV and Pan American Health Organization, which estimates the costs and health outcomes of PMTCT interventions [20]. Costs of ARVs and laboratory tests including rapid HIV test were estimated based on the WHO Global Price Reporting Mechanism as well as on the Clinton Health Access Initiative (CHAI) price list [21], [22], [23]. Costs of health services were derived from the WHO Choosing Interventions that are Cost Effective (WHO-CHOICE) [24]. All costs were discounted at 3% annually. Costs of PMTCT included HIV testing for both pregnant women and their partners, ARVs, other necessary laboratory tests, and health care services. Treatment cost included the first line ARVs, laboratory monitoring, and health care services for out-patient clinic.

### Main Outcomes

Main outcomes of this study are divided into two categories, namely health related outcomes and cost related outcomes. In health related outcomes, expected risks and number of HIV transmission to infants at the age of 18 months, HIV transmission to serodiscordant partners, and ART initiation of HIV-infected pregnant women with CD4 cell count of ≤350 cells/mm^3^ were examined and compared among 2010 Option A, ARVs during pregnancy and breastfeeding (Option B), and Lifelong ART (Option B+). Estimated probabilities of transmission to infants and serodiscordant partners and ART initiation by the model were entered into the CTEI, which provided the estimated number of infections par annual cohort of pregnant women in Zambia.

As for cost related outcomes, total costs of PMTCT services per annual cohort of pregnant women in the country as well as future treatment costs as a result of HIV transmission to infants and serodiscordant partners over a period of ten years were examined and compared among different options. Incremental cost-effectiveness ratio (ICER) with regards to infant and partner infections averted and quality-adjusted life-year (QALY) gained were calculated. In this study we applied 16.88 QALYs gained per infant infection averted [25] and 5.83 QALYs gained per partner infection averted [26] based on the past study.

### Analysis

First, base-case analysis was conducted, in which we examined outcomes of each options under the current health service coverage and utilization in Zambia. Then we performed sensitivity analysis on key parameters in order to examine the robustness of our findings. Parameters including access and utilization of health services, discordance rate, and HIV prevalence were varied enhancing their possible range as well as the changes in the future (e.g. improvement in service coverage and reduction of HIV prevalence).

Cost-effectiveness analysis was conducted, in which cost per infection averted and ICER per QALY gained were calculated. Based on the WHO’s guidance, we defined that ICER below the annual gross domestic product (GDP) per capita in the country as very cost-effective, and below the three times of GDP per capita as cost-effective [27].

## Results

### Base-case Analysis

**Figure 2:**
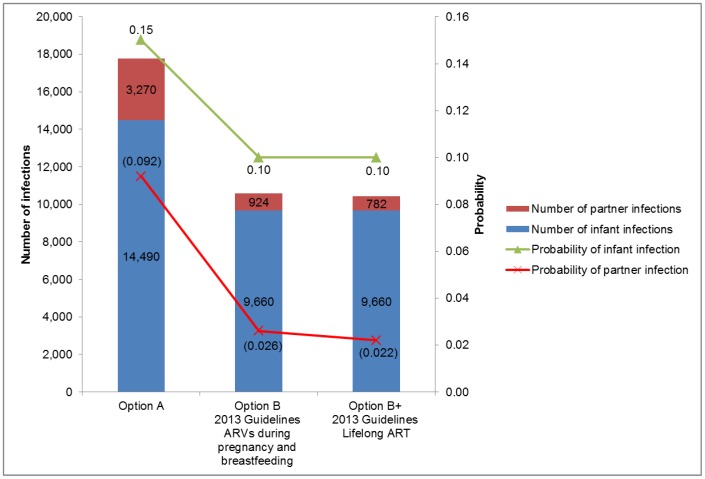
Health outcomes - HIV infections among exposed infants and serodiscordant partners.

The results of base-case analysis for health related outcomes on HIV transmission are presented in Figure 2. With current PMTCT services coverage and assuming 12 months of breastfeeding, the estimated probability of perinatal HIV infection was 0.15 for 2010 Option A and 0.10 for ARVs during pregnancy and breastfeeding (Option B) and Lifelong ART (Option B+). These rates applied to the annual cohort of 600,000 pregnant women with HIV prevalence of 16.1% (i.e. 96,600 HIV-infected pregnant women) would result in a total of 14,490 new infections among exposed infants when applying 2010 Option A and 9,660 infections in the case of 2013 guidelines (Option B and B+). This indicates that the transmission risk for HIV exposed infants will be further reduced by 33% through shifting from 2010 Option A to 2013 guidelines within the current service coverage.

With respect to HIV transmission to serodiscordant partners, the estimated probability of infection was 0.092 for 2010 Option A, 0.026 for Option B, and 0.022 for Option B+ over a period of 24 months (i.e. from enrolment to PMTCT services to 18 months postnatal). This equals to a total of 3,270 new infections among serodiscordant partners per annual cohort of 600,000 pregnant women for Option A, 924 infections for Option B, and 782 infections for Option B+. That would represent a 72% reduction in the risk of transmission to serodiscordant partners following a shift from Option A to Option B, while a further reduction of 15% could be expected when shifting from Option B to Option B+.

**Figure 3:**
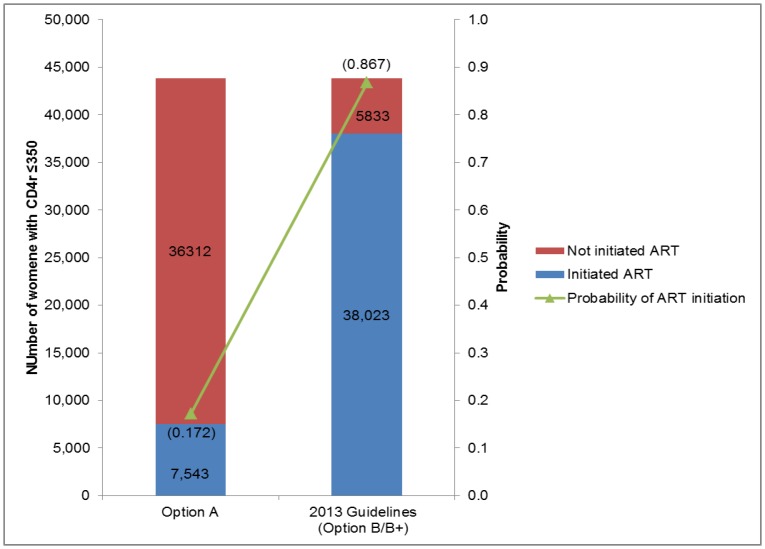
ART initiation of HIV-infected pregnant women with CD4 cell count of ≤350 cells/mm^3^.

Probability of ART initiation for HIV-infected pregnant women with CD4 cell count of ≤350 cells/mm^3^ was 0.172 for 2010 Option A and 0.867 for 2013 guidelines with the current level of service access and utilization (Figure 3). As a result, more than 80% of women with CD4 cell count of ≤350 cells/mm^3^ would fail to timely initiate ART within the Option A scenario, whereas the proportion would drop to 13% when applying the 2013 guidelines.

Table 2 shows the results of base-case analysis on cost related outcomes. The total cost of PMTCT services from enrolment to 18 months postnatal at current levels of access and utilization of PMTCT services was 16,251,561 USD for Option A per annual cohort of 600,000 pregnant women, 23,415,954 USD for Option B, and 28,884,860 USD for Option B+. Cost per infant infection averted for Option A was 1,034 USD, 1,140 USD for Option B, and 1,406 USD for Option B+. When prevention of partner infections was also included, the cost per infection averted was reduced to 1,023 USD for Option B and 1,254 USD for Option B+. The incremental cost-effectiveness ratio (ICER) per QALY gained by averting infant infection was 88 USD for Option B and 155 USD for Option B+; the incremental cost per QALY gained for both infant and partner was 75 USD for Option B and 132 USD for Option B+. As these were well below the annual GDP per capita of 1,469 USD in Zambia in 2012 [28], Option B and B+ were found very cost-effective, and more so when partner infections were taken into account.

**Table 2:** Cost related outcomes - Base-case analysis.

		2010 guidelines	2013 guidelines	
		Option A	Option B	Option B+
Costs (USD)				
Costs of PMTCT programme^a^		16,251,561	23,415,954	28,884,860
10 years treatment costs				
Infected children		27,002,616	18,001,744	18,001,744
Infected partners		7,751,060	2,190,517	1,853,514
Total costs		51,005,237	43,608,215	48,740,118
Number of infections				
Infants		14,490	9,660	9,660
Partners		3,270	924	782
Total		17,760	10,584	10,442
Cost per infection averted^b^ (USD)				
Infant infection only		1,034	1,140	1,406
Infant and partner infection		1,034	1,023	1,254
QALYs gained (compared to Option A)				
Infants		–	81,530	81,530
Partners		–	13,677	14,505
Total		–	95,208	96,035
ICER per QALY gained (USD)				
Based on costs of PMTCT programme				
Infant infection only		–	88	155
Infant and partner infections		–	75	132
Based on costs of PMTCT programme+future treatment costs				
Infant infection only		–	Dominant	Dominant
Infant and partner infections		–	Dominant	Dominant

a For a period of 24 months (from 14 weeks of pregnancy to 18 months after delivery) per annual cohort of 600,000 pregnant women.

b Based on the costs of PMTCT programme.

**Figure 4:**
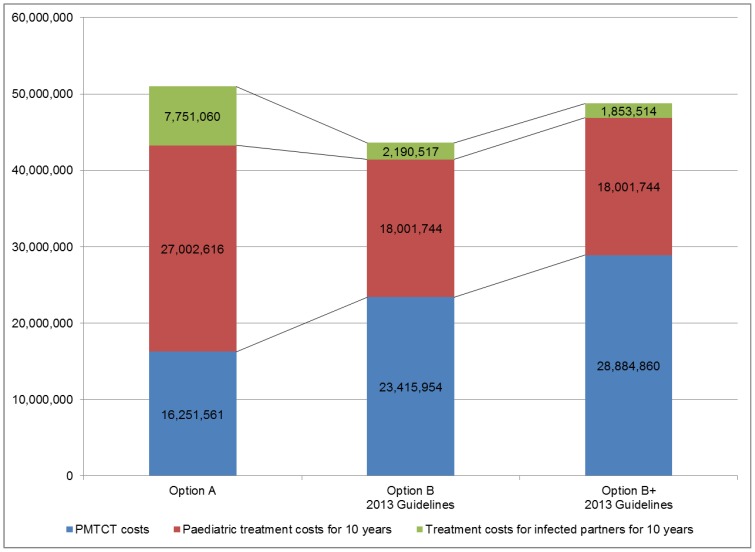
PMTCT costs and future treatment costs of infected infants and partners for 10 years (USD).

We estimated treatment costs over a period of 10 years for infected children and partners and examined the long term impact of averted infections on overall costs: costs of PMTCT services and future treatment costs as a result of HIV transmission (Figure 4). As Option B is estimated to avert an additional 4,830 infant infections and 2,346 infections among serodiscordant couples compared to Option A, it would cost 7,397,022 USD less than Option A due to the reduction of future treatment costs. Option B+ is estimated to avert 2,488 additional infections among partners and it would cost 2,265,119 USD less compared to Option A. Then ICERs per QALY gained based on total costs of PMTCT programme and future treatment costs were calculated (Table 2). The 2013 guidelines, both Option B and B+ were dominant compared to Option A.

### Sensitivity Analysis

Key parameters, including access and utilization of health services, discordance rate, and HIV prevalence were varied and their impact was examined (Table 3).

**Table 3:** Sensitivity analysis.

	Health outcomes			Total costs^a^	QALYs gained^b^	ICER per QALY gained (USD)	
	Infant infections	Partner infections	ART initiation	(USD)	vs Option A	vs Option A	vs Option B
**Access and utilization of services**							
Base-case							
Option A	14,490	3,270	7,543	51,005,237	–	–	–
Option B	9,660	924	38,023	43,608,215	95,208	Dominant	-
Option B+	9,660	782	38,023	48,740,118	96,035	Dominant	6,199
Best-case (90% coverage)							
Option A	6,762	1,564	31,975	35,483,008	–	–	–
Option B	5,796	462	43,422	39,679,813	22,731	185	–
Option B+	5,796	320	43,422	45,914,259	23,559	443	7,531
**Discordance rate (HIV+ women with HIV- partners)**							
40%							
Option A	14,490	3,555	7,543	51,679,527	–	–	–
Option B	9,660	1,005	38,023	43,799,548	96,397	Dominant	-
Option B+	9,660	850	38,023	48,901,104	97,301	Dominant	5,645
5%							
Option A	14,490	444	7,543	44,306,457	–	–	–
Option B	9,660	126	38,023	41,716,318	83,384	Dominant	–
Option B+	9,660	106	38,023	47,137,824	83,501	34	46,497
**HIV prevalence (women aged 15–49)**							
20%							
Option A	18,000	4,063	9,371	63,103,727	–	–	–
Option B	12,000	1,148	47,234	53,913,837	118,274	Dominant	-
Option B+	12,000	972	47,234	60,290,389	119,301	Dominant	6,214
1%							
Option A	900	203	469	4,164,611	–	–	–
Option B	600	57	2,362	3,704,524	5,915	Dominant	–
Option B+	600	49	2,362	4,025,248	5,962	Dominant	6,877

a Total costs = PMTCT programme costs+future treatment costs of infected children and partners for 10 years.

b QALYs gained by averting infant and partner infections.

#### Access and utilization of health services

Probability of access to CD4 test, receipt of its result, initiation of maternal ARV prophylaxis, initiation of ART for women with CD4 cell count of ≤350 cells/mm^3^, and initiation of infant NVP prophylaxis were increased to 0.9 (i.e. Best-case) and entered into the model. The differences in outcomes among the options were reduced, especially in terms of infant infection and ART initiation of women with CD4 cell count of ≤350 cells/mm^3^. The probability of transmission to infant was 0.07 for Option A and 0.06 for 2013 guidelines. The estimated number of infant infection decreased in all options, with a total of 6,762 infections expected for Option A and 5,796 infections for 2013 guidelines. The risk and number of infections to discordant partners also decreased, with 1,564 infections (probability 0.044) expected for Option A, 462 infections (probability 0.013) for Option B, and 320 infections (probability 0.009) for Option B+. Number of women initiating ART increased to 31,975 (72.9% of all women with CD4 cell count of ≤350 cells/mm^3^) for Option A and to 43,422 (99%) for 2013 guidelines. Although ICER per QALY gained increased to 185 USD for Option B and 443 for Option B+, they still remained very cost-effective (i.e. well below the annual GDP per capita).

#### Discordance rate

We varied the ratio of HIV-infected pregnant women with seronegative partners and examined its impact on outcomes. It was found that the higher the discordance rate, the more cost-effective the outcomes. For example, the number of expected infections among discordant partners was ranging between 444 to 3,555 for Option A, 126 to 1,005 for Option B, and 106 to 850 for Option B+. In all scenarios, Option B was dominant compared to Option A. Option B+ was dominant with discordance rate of 40% and very cost-effective with discordance rate of 5%. When Option B and B+ were compared, ICER per QALY gained ranged between 5,645 to 45,497 USD.

#### HIV prevalence

HIV prevalence among pregnant women was varied between 1% and 20% in order to see the implication of future declines in HIV prevalence among women. The number of expected infant infections ranged between 900 and 18,000 for Option A and between 600 and 12,000 for the 2013 guidelines. The expected number of infection among partners ranged between 203 and 4,063 for Option A, 57 and 1,148 for Option B, and 49 and 972 for Option B+. The 2013 guidelines were dominant compared to Option A and remained so across different prevalence rates even with a low HIV prevalence of 1%. When Option B+ was compared to Option B, the ICER ranged between 6,214 to 6,877 USD, which was 4.2 to 4.7 times of the annual GDP per capita.

#### Treatment costs for infected infants and partners

We also conducted break-even analysis varying the cost of treatment in order to identify break-even points of treatment costs for infected children and partners against costs of PMTCT services (i.e. Incremental costs of PMTCT services  = 10 years costs of treatment for infants and partners averted by PMTCT). For this analysis we assumed the same treatment costs for both adult and children. Break-even point was 998 USD for Option B against Option A and 1,726 USD for Option B+ against Option A. In other words, Option B will be cost-saving if the cost of treatment for 10 years is more than 998 USD per patient; Option B+ will be cost-saving if the treatment cost for 10 years is more than 1,726 USD.

## Discussion

This study examined health outcomes and cost impact of the shift to WHO 2013 recommendations in the context of Zambia. It examined the differences between WHO 2010 guidelines Option A and new WHO 2013 guidelines in terms of their effectiveness in preventing HIV transmission to infants and to serodiscordant partners and initiating ART for HIV-infected women with CD4 cell count of ≤350 cells/mm^3^.

Similar studies conducted in the past examined the cost-effectiveness of Option A and Option B with regards to the health outcomes of exposed children. Some have also examined Option B+ as well as maternal health outcomes. Studies conducted in Tanzania, Malawi, and Nigeria have examined the cost-effectiveness of highly active antiretroviral therapy (HAART) for PMTCT compared to no intervention and/or previous guidelines including single dose NVP, and concluded that HAART was a cost-effective intervention in resource-limited settings [29], [30], [31]. Recent studies conducted in Uganda, Rwanda, Zimbabwe, Malawi, and South Africa examined the cost-effectiveness of WHO 2010 guidelines with regards to health outcomes in exposed infants, while some also examined the health outcomes of mothers, and their results supported the shift from their current practices to Option B/B+ [32], [33], [34], [35], [36].

To our knowledge, this is the first study to examine the new WHO 2013 guidelines and the expanded impact of PMTCT interventions on preventing HIV infection in serodiscordant partners. We would like to highlight four main findings from this study, which not only support the conclusions of previous studies, but also provide additional evidence of the benefit of new WHO 2013 guidelines.

First of all, our study showed that the 2013 guidelines improve health outcomes of exposed infants, HIV infected pregnant women, and serodiscordant partners compared to WHO 2010 guidelines Option A. Our analysis indicated that the transmission risk for HIV exposed infants could be further reduced by 33% through shifting from 2010 Option A to 2013 guidelines with current service coverage. One of the main factors for this reduction would be the increased access to ART among HIV-infected pregnant women with CD4 cell count of ≤350 cells/mm^3^ by removing the barrier of CD4 testing as a pre-requisite for ART initiation. Another factor in favour of the 2013 guidelines would be the higher maternal ARV prophylaxis coverage achieved during the breastfeeding period compared to 2010 Option A. Field experiences of the implementation of 2010 Option A in rural areas in Zambia have shown several challenges with the infant NVP prophylaxis in exposed infants, including dosage adjustment and retention of exposed infants to PMTCT services [37]. A modelling study has also indicated the higher risk of postnatal HIV transmission of 2010 Option A compared to Option B at the field level [38]. These findings document that the provision of ARVs to mothers rather than infants is more feasible and it could improve adherence to drugs during breastfeeding period thus resulting in reduced risk of transmission. Furthermore, when we take into account the impact on subsequent pregnancies among HIV-infected women who are already on ART, the transmission risk would be reduced further and the risk difference between 2010 Option A and 2013 guidelines could grow wider.

The shift from Option A to Option B would result in a 72% reduction in the risk of transmission to serodiscordant partners. This large scale reduction represents a strong benefit expected from 2013 guidelines. Provision of ART for all pregnant women living with HIV offers additional advantages in settings of high HIV prevalence where coverage of HIV testing among partners is low. In fact, our study shows that the shift from Option B to Option B+, which adds another 6 months of ART for women living with HIV in our model, will result in a further 15% reduction of transmission risk. When a longer period of time was considered, the model suggested further reduction of the risk of transmission to serodiscordant partners in Option B+.

PMTCT services have been recognized as an important entry point to HIV care and treatment services for women living with HIV. However, because of several bottle necks in the service delivery system mainly related to CD4 testing including access to services and return of results, only a limited proportion of women actually initiate treatment [39]. Our study showed that this missed opportunity for HIV-infected pregnant women with CD4 cell count of ≤350 cells/mm^3^ would be significantly reduced by shifting from 2010 Option A to 2013 guidelines.

Second, 2013 guidelines are very cost-effective compared to Option A and also save costs related to future care and treatment needs. With regard to the PMTCT programme costs, when prevention of partner infections was taken into account in addition to infant infections, the cost-effectiveness of 2013 guidelines further improved. When we consider the future costs of the treatment of infected infants and partners over a period of 10 years, the 2013 guidelines were found to be cost-saving compared to Option A, in which Option B would save 7.4 million USD and Option B+ would save 2.3 million USD compared to Option A per annual cohort of pregnant women. It should be noted that the estimated cost-savings in this study may be conservative as we only applied the minimum cost of ARV treatment and not the costs of opportunistic infections and hospitalizations. Therefore, the savings could be higher if these costs were also considered.

Third, the cost-effectiveness of the 2013 guidelines is robust even in a scenario of low HIV prevalence. Our sensitivity analysis showed that even with the HIV prevalence of 1%, Option B and B+ remained cost-effective. This finding has an implication not only for the reduced prevalence for the future, but also for the other areas and countries with low HIV prevalence and support the shift to the 2013 guidelines in such settings.

Forth, Option B+ is found less cost-effective compared to Option B in our model. The ICER of Option B+ compared to Option B was above the three times of the annual GDP per capita, and remained so in the sensitivity analysis. Main reason for this may be the time horizon for this analysis. As we have assumed 12 months of breastfeeding and estimated the health impact of each option at 18 months after delivery, the difference of the period of ART for HIV-infected women between Option B and B+ was 6 months only, which may resulted in a relatively limited impact on reducing HIV transmission to serodiscordant partners. Moreover, while we estimated QALYs gained for both infants and partners in our model, if maternal health gains were also included, the cost-effectiveness of Option B+ would be further improved as shown in the studies in Malawi and Zimbabwe [34], [35].

In summary, our findings favour the WHO 2013 guidelines and support the shift from 2010 Option A to Option B and Option B+ in Zambia. The benefit of lifelong ART or 3ARVs given to HIV-infected pregnant and breastfeeding women is demonstrated in this study, in which impact is not only limited to prevention of infant infection, but also extended to the prevention of transmission to serodiscordant partners as well as ART initiation for women.

Field implementation of the 2013 guidelines would require an initial investment in PMTCT services as well as strengthened health system, especially in remote areas [6], [11], [40]. For example, it is necessary to increase the number of health facilities those are able to provide ART or 3ARVs which has been provided only at large health facilities in the past. Capacity building of healthcare workers working for maternal and child health care services who are not familiar with providing ART is needed, which require trainings and regular supervisions. Supply management system needs to be strengthened to provide ARVs, test kits, and other materials to each health centre which provides PMTCT and ART services. Adherence and retention to ART is another important issue, where strong linkages and/or integration of maternal and child health services and HIV services as well as enhanced care and support for patients need to be ensured. ARV toxicity and possible development of drug resistance are other areas of concern and should be closely monitored through an enhanced surveillance system and pharmacovigilance programme. The early experiences of Option B+ implementation in Malawi have demonstrated some of these challenges [41]. It is possible that inadequate investment and preparations together with weak health system may hamper the expected impact of the 2013 guidelines. As many countries are now shifting to the 2013 guidelines, there is a need to closely monitor and evaluate the implementation of Option B and B+.

This study has several limitations. First, we may have underestimated the impact of 2010 Option A in terms of preventing infection among serodiscordant couples as we assumed that ZDV alone would not prevent transmission of HIV to negative partners. Second, while we have applied the transmission risks among heterosexual couples based on the past studies, the actual risk of transmission from pregnant women to partners may differ due to the biological and behavioural factors. In addition, since our model has set CD4 cell count of ≤350 cells/mm^3^ as a priority for lifelong ART, this may have influenced the transmission risk to serodiscordant partners in Option B. Third, neither the impact of earlier initiation of ART in improving the health of HIV-positive mothers and its positive effect on her children’s health, nor the prevention benefits in subsequent pregnancies were reflected in the study, resulting in a possible underestimation of the potential impact of 2013 guidelines. Forth, as we did not include the costs of second/third line regimens for ART, treatment of opportunistic infections (OIs), and hospitalization, we may have underestimated the total costs of treatment. The methods of estimating treatment costs varied in other similar studies; some included second line regimen but not OIs treatment [33], [36]. However, it is expected that by considering the health outcome of women and including other related costs of treatment, it would further improve the cost-effectiveness of 2013 guidelines, in particular Option B+. Fifth, the difference between Option B and B+ in our model was relatively small, in which women in Option B+ received 6 months additional ART compared to Option B, which may have limited the overall health impact of Option B+. The findings of this study should be interpreted considering these limitations and further research addressing these issues is needed.

## Conclusion

Our findings support the shift to new WHO 2013 guidelines in Zambia, as they would improve health of family through preventing HIV transmission to infants, enhancing health of mothers, and preventing transmission of HIV infection to serodiscordant partners and save future costs related to care and treatment.

## References

[1] De Cock KM, Fowler MG, Mercier E, de Vincenzi I, Saba J, et al (2000) Prevention of mother-to-child HIV transmission in resource-poor countries: translating research into policy and practice. JAMA 283: 1175–1182. 10703780 10.1001/jama.283.9.1175

[2] WHO (2010) Antiretroviral drugs for treating pregnant women and preventing HIV infections in infants: recommendations for a public health approach. Geneva, Switzerland: WHO. 26180894

[3] UNAIDS (2011) Global plan towards the elimination of new HIV infections among children by 2015 and keeping their mothers alive. Geneva: UNAIDS.

[4] UNAIDS (2013) Global report: UNAIDS report on the global AIDS epidemic 2013. Geneva: UNAIDS.

[5] WHO (2012) Programmatic update: use of antiretroviral drugs for treating pregnant women and preventing HIV infection in infants. Geneva, Switzerland: WHO.

[6] WHO (2013) Consolidated guidelines on the use of antiretroviral drugs for treating and preventing HIV infection: recommendations for a public health approach. Geneva, Switzerland: WHO. 24716260

[7] Mugo NR, Med M, Heffron R, Donnell D, Wald A, et al (2011) Increased risk of HIV-1 transmission in pregnancy: a prospective study among African HIV-1 serodiscordant couples. AIDS 25: 1887–1895. 21785321 10.1097/QAD.0b013e32834a9338 PMC3173565

[8] Cohen MS, Chen YQ, McCauley M, Gamble T, Hosseinipour MC, et al (2011) Prevention of HIV-1 infection with early antiretroviral therapy. N Engl J Med 365: 493–505. 21767103 10.1056/NEJMoa1105243 PMC3200068

[9] Marazzi MC, Palombi L, Nielsen-Saines K, Haswell J, Zimba I, et al (2011) Extended antenatal use of triple antiretroviral therapy for prevention of mother-to-child transmission of HIV-1 correlates with favorable pregnancy outcomes. AIDS 25: 1611–1618. 21673553 10.1097/QAD.0b013e3283493ed0

[10] Ministry of Health Zambia (2012) Zambia country report: monitoring the declaration of committment on HIV and AIDS and the Universal Access. Lusaka: Ministry of Health Zambia.

[11] Zambia (2012) Business case for an improved eMTCT protocol in Zambia - Moving to Option B+. Lusaka, Zambia: Available: http://emtct-iatt-v2.org.php54-1.dfw1-2.websitetestlink.com/wp-content/uploads/2013/02/Zambia-B+-Business-Case-Jan132.pdf Accessed 2013 Dec 28.

[12] UNAIDS Reference Group on Estimates Modelling and Projections (2011) Working Paper on Mother-to-Child HIV Transmission Rates for use in Spectrum.

[13] Donnell D, Baeten JM, Kiarie J, Thomas KK, Stevens W, et al (2010) Heterosexual HIV-1 transmission after initiation of antiretroviral therapy: a prospective cohort analysis. Lancet 375: 2092–2098. 20537376 10.1016/S0140-6736(10)60705-2 PMC2922041

[14] Hallett TB, Baeten JM, Heffron R, Barnabas R, de Bruyn G, et al (2011) Optimal uses of antiretrovirals for prevention in HIV-1 serodiscordant heterosexual couples in South Africa: a modelling study. PLoS Med 8: e1001123. 22110407 10.1371/journal.pmed.1001123 PMC3217021

[15] Reynolds SJ, Makumbi F, Nakigozi G, Kagaayi J, Gray RH, et al (2011) HIV-1 transmission among HIV-1 discordant couples before and after the introduction of antiretroviral therapy. AIDS 25: 473–477. 21160416 10.1097/QAD.0b013e3283437c2b PMC3261071

[16] Attia S, Egger M, Muller M, Zwahlen M, Low N (2009) Sexual transmission of HIV according to viral load and antiretroviral therapy: systematic review and meta-analysis. AIDS 23: 1397–1404. 19381076 10.1097/QAD.0b013e32832b7dca

[17] Carter RJ, Dugan K, El-Sadr WM, Myer L, Otieno J, et al (2010) CD4+ cell count testing more effective than HIV disease clinical staging in identifying pregnant and postpartum women eligible for antiretroviral therapy in resource-limited settings. J Acquir Immune Defic Syndr 55: 404–410. 20595905 10.1097/QAI.0b013e3181e73f4b

[18] Central Statistical Office, Ministry of Health Zambia, Tropical Diseases Research Centre, University of Zambia, Macro International Inc (2009) Zambia Demographic and Health Survey 2007. Maryland, USA: CSO and Macro International Inc.

[19] United Nations (2010) UN Data. Available: http://data.un.org/Search.aspx?q=annual+number+of+birth Accessed 2012 Sep 15.

[20] Ishikawa N, Shimbo T, Miyano S (2011) Costing Tool for Elimination Initiative (CTEI) From costing to planning: a tool to support the initiative for elimination of new paediatric HIV infections and congenital syphilis and improvement of the health and survival of mothers and infants. National Center for Global Health and Medicine (NCGM), Asia-Pacific United Nations Task Force for the Prevention of Parents-to-Child Transmission of HIV, and Pan American Health Organization (PAHO): Available: http://www.eptctasiapacific.org/funding-resource-needs.

[21] Clinton Health Access Initiative (2012) Antiretroviral (ARV) ceiling price list. Clinton Health Access Initiative: Available: http://www.clintonhealthaccess.org/news-and-information/ARV-Ceiling-Price-List-May-2012 Accessed 2012 Sep 10.

[22] WHO Global price reporting mechanism. Geneva: Available: http://www.who.int/hiv/amds/gprm/en/index.html. Accessed 2012 Sep 10.

[23] Clinton Foundation (2009) HIV/AIDS diagnostic pricing outlook. Available: http://www.who.int/hiv/topics/treatment/costing_clinton_diagnostic.pdf. Accessed 2012 Aug 15.

[24] WHO Choosing interventions that are cost effective (WHO-CHOICE). Geneva: Available: http://www.who.int/choice/costs/en/index.html Accessed 2012 Sep 17.

[25] Soorapanth S, Sansom S, Bulterys M, Besser M, Theron G, et al (2006) Cost-effectiveness of HIV rescreening during late pregnancy to prevent mother-to-child HIV transmission in South Africa and other resource-limited settings. J Acquir Immune Defic Syndr 42: 213–221. 16639346 10.1097/01.qai.0000214812.72916.bc

[26] Farnham PG, Gopalappa C, Sansom SL, Hutchinson AB, Brooks JT, et al (2013) Updates of lifetime costs of care and quality-of-life estimates for HIV-infected persons in the United States: late versus early diagnosis and entry into care. J Acquir Immune Defic Syndr 64: 183–189. 23615000 10.1097/QAI.0b013e3182973966

[27] WHO (2003) Guide to cost-effectiveness analysis. Geneva.

[28] World Bank (2013) GDP per capita (current US$). Washington DC: Available: http://data.worldbank.org/indicator/NY.GDP.PCAP.CD Accessed 2013 Dec 5.

[29] Robberstad B, Evjen-Olsen B (2010) Preventing mother to child transmission of HIV with highly active antiretroviral treatment in Tanzania–a prospective cost-effectiveness study. J Acquir Immune Defic Syndr 55: 397–403. 20739897 10.1097/QAI.0b013e3181eef4d3

[30] Orlando S, Marazzi MC, Mancinelli S, Liotta G, Ceffa S, et al (2010) Cost-effectiveness of using HAART in prevention of mother-to-child transmission in the DREAM-Project Malawi. J Acquir Immune Defic Syndr 55: 631–634. 21934555 10.1097/QAI.0b013e3181f9f9f5

[31] Shah M, Johns B, Abimiku A, Walker DG (2011) Cost-effectiveness of new WHO recommendations for prevention of mother-to-child transmission of HIV in a resource-limited setting. AIDS 25: 1093–1102. 21505317 10.1097/QAD.0b013e32834670b9

[32] Kuznik A, Lamorde M, Hermans S, Castelnuovo B, Auerbach B, et al (2012) Evaluating the cost-effectiveness of combination antiretroviral therapy for the prevention of mother-to-child transmission of HIV in Uganda. Bull World Health Organ 90: 595–603. 22893743 10.2471/BLT.11.095430 PMC3417786

[33] Binagwaho A, Pegurri E, Drobac PC, Mugwaneza P, Stulac SN, et al (2013) Prevention of Mother-To-Child Transmission of HIV: Cost-Effectiveness of Antiretroviral Regimens and Feeding Options in Rwanda. PLoS One 8: e54180. 23437040 10.1371/journal.pone.0054180 PMC3577801

[34] Ciaranello AL, Perez F, Engelsmann B, Walensky RP, Mushavi A, et al (2013) Cost-effectiveness of World Health Organization 2010 guidelines for prevention of mother-to-child HIV transmission in Zimbabwe. Clin Infect Dis 56: 430–446. 23204035 10.1093/cid/cis858 PMC3540037

[35] Fasawe O, Avila C, Shaffer N, Schouten E, Chimbwandira F, et al (2013) Cost-effectiveness analysis of Option B+ for HIV prevention and treatment of mothers and children in Malawi. PLoS One 8: e57778. 23554867 10.1371/journal.pone.0057778 PMC3595266

[36] Zulliger R, Black S, Holtgrave DR, Ciaranello AL, Bekker LG, et al. (2013) Cost-Effectiveness of a Package of Interventions for Expedited Antiretroviral Therapy Initiation During Pregnancy in Cape Town, South Africa. AIDS Behav. 10.1007/s10461-013-0641-7 PMC3984926 24122044

[37] Changala M, Kapyata H, Kahula M, Siachiwena C, Kalichini P, et al (2012) Mothers’ confusions over the extended nevirapine regimen for HIV-exposed infants in resource-limited settings. 22–27 July 2012, XIX International AIDS Conference (Abstract LBPE30; http://wwwiasocietyorg/Defaultaspx?pageId=11&abstractId=200747587). Washington DC, USA.

[38] Ishikawa N, Shimbo T, Miyano S, Sikazwe I, Ghidinelli MN, et al (2012) Field effectiveness of WHO PMTCT guidelines in preventing postnatal HIV transmission in resource-limited settings: operational barriers and complexities related to the implementation of extended infant prophylaxis. 22–27 July 2012, XIX International AIDS Conference (Abstract TUPE177; http://wwwiasocietyorg/Defaultaspx?pageId=11&abstractId=200744282). Washington DC, USA.

[39] Ishikawa N, Miyano S, Shimbo T (2011) Treating all HIV-infected pregnant women irrespective of CD4 count: missed opportunity for ART initiation during the prevention of mother-to-child transmission of HIV. 17–20 July 2011, 6th IAS Conference on HIV Pathogenesis and Treatment, (Abstract TULBPE056; http://wwwiasocietyorg/Defaultaspx?pageId=11&abstractId=200743928). Roma, Italy.

[40] The Interagency Task Team (2013) Toolkit, Expanding and Simplifying Treatment for Pregnant Women Living with HIV: Managing the Transition to Option B/B+.

[41] UNICEF (2013) Rapid Assessment of the 2010 WHO Guidelines in Four Countries: Lesotho, Malawi, the United Republic of Tanzania and Zambia. New York: UNICEF.

